# Motor cortex excitability during spine shape-judgment in adolescent idiopathic scoliosis: a TMS motor evoked potential study

**DOI:** 10.1007/s00221-026-07343-5

**Published:** 2026-06-30

**Authors:** Mateja Virovec, Roberto Barumerli, Paola Cesari

**Affiliations:** 1 Department of Neuroscience, Biomedicine and Movement Sciences, University of Verona, Via Casorati 43, Verona, 37131 Italy; 2 Dyson School of Design Engineering, Imperial College London, London, UK

**Keywords:** Body representation, Adolescent idiopathic scoliosis, Corticospinal excitability, Transcranial magnetic stimulation, Motor evoked potentials

## Abstract

This study investigated the neurophysiological mechanisms linking visual body perception to motor output in females with adolescent idiopathic scoliosis (AIS), specifically examining how altered body schema influences corticospinal excitability. A two-stage paradigm was employed in participants with AIS and healthy controls. First, psychophysical thresholds for detecting spinal curvature were estimated. Second, single-pulse transcranial magnetic stimulation (TMS) was applied over the primary motor cortex (100–125 ms post-stimulus) to assess motor system reactivity to body-relevant stimuli. Corticospinal excitability was recorded via motor evoked potentials (MEPs) from intrinsic hand muscles as a proxy for motor readiness during the observation of spinal distortions. Results revealed that AIS patients exhibited significantly lower perceptual thresholds than controls, indicating hypersensitivity to spinal curvature that correlated with subjective self-perception (Trunk Appearance Perception Scale; TAPS). TAPS scores did not correlate significantly with participants’ Cobb angles, indicating that subjective body schema distortion operates independently of mechanical deformity severity. In addition, TMS revealed that AIS patients reached peak corticospinal excitability at their lower perceptual threshold, whereas healthy controls demonstrated peak excitability only when spinal curvatures exceeded their detection thresholds. These findings suggest that AIS involves an increased corticospinal gain in response to body-relevant stimuli. Rather than serving purely as a detection mechanism, the motor system in AIS exhibits a heightened readiness that is tightly coupled with the perception of spinal distortions. Such sensorimotor reorganization suggests that motor excitability is adaptively recalibrated to the patient’s lived physical experience.

**Supplementary Information:**

The online version contains supplementary material available at 10.1007/s00221-026-07343-5.

## Introduction

Body representation is a multidimensional construct emerging from the continuous integration of visual, proprioceptive, tactile, vestibular, and interoceptive signals, enabling the brain to maintain an up-to-date model of the body’s position and shape (Palermo et al. 2014). This multisensory integration underpins the body schema, an unconscious sensorimotor model of our body that allows accurate motor planning and execution (Gallagher 1986). On the other hand, the conscious body image originated from the same inputs, shapes self-awareness, and emotional, behavioral, and perceptive attitudes toward one’s own body (Hosseini and Padhy 2023). Accurate body perception is consequently essential for coordinated movement, for maintaining a stable self-image, and for successful social interaction, as disturbances in any sensory route can alter body ownership and impair interpersonal cognition (Tsakiris 2017). Adolescent Idiopathic Scoliosis (AIS), primarily viewed as an orthopedic disorder, frequently presents sensory-perceptual disturbances, suggesting a deeper neurological involvement (Herman et al. 1985).

AIS, also known as late-onset scoliosis that typically emerges during the adolescent growth spurt, is characterized by a three-dimensional lateral curvature of the spine in the coronal plane (Weinstein et al. 2008). The level of deformation is measured using the Cobb angle (Cobb 1948), which represents the angle between the endplates of the upper and lower end vertebrae. The scoliosis is confirmed if the Cobb angle exceeds 10 degrees as measured by x-rays (Gstoettner et al. 2007). AIS is twice as prevalent in girls as in boys, rising to 3:1 in the age group of 11–12 years (Cili et al., 2009). Beyond physical curvature, girls with AIS often suffer from trunk misalignment and dysfunctional neuromuscular signaling, which may stem from a disrupted body schema (Picelli et al. 2016). Specifically, individuals with AIS exhibit impaired standing balance and a deficit in integrating body orientation cues (Le Berre et al. 2019). Recent evidence suggests that these patients possess a specific attentional bias toward affected body parts. Bertuccelli and colleagues (2023) reported faster response times and higher accuracy in AIS patients when processing stimuli related to the back and shoulders. This suggests that the persistent need to monitor spinal asymmetry leads to a heightened neural prioritization of body-relevant stimuli.

Furthermore, neuroimaging and neurophysiological studies support a central nervous system (CNS) origin for these deficits, revealing altered white-matter pathways (Paramento et al. 2023) and functional changes in the supplementary motor area (SMA) and posterior parietal cortex (PPC), which are the key areas responsible for body schema integration (Formaggio et al. 2022). Previous TMS studies have identified baseline hyperexcitability and reduced intracortical inhibition in the motor cortex of AIS patients (Doménech et al. 2010; Boček et al. 2022). These findings indicate an increased corticospinal gain, likely resulting from chronic adaptive responses to spinal deformity. However, while we know that AIS motor cortex is baseline-hyperexcitable, a clear mechanism linking this neural ‘readiness’ to the visual processing of body-specific deformity remains unexplored. Specifically, it remains undefined to what extent this heightened excitability is a consequence of pathology versus a functional mechanism directing how patients perceive and react to body-related visual information.

Prior research has demonstrated that observing body-related stimuli modulates motor cortex excitability through embodied simulation, a process that links visual perception to the motor system. Viewing the human body is not a passive visual process, instead, it activates the observer’s primary motor cortex (M1), reflecting the integration of visual information into the internal body schema (Fadiga et al. 2005). Within this framework, motivationally salient or self-relevant images can significantly enhance corticospinal gain (Borgomaneri et al. 2021). Because M1 receives dense projections from posterior parietal regions involved in body schema maintenance (Rizzolatti and Luppino 2001), and distal hand control is functionally coupled with trunk representation, hand MEPs serve as a sensitive proxy for the overall state of the sensorimotor network (Chiou et al. 2016).

The present study employed a two-stage paradigm to investigate the relationship between perceptual sensitivity and motor cortex excitability in females with AIS and healthy matched controls. In the first task, we used a psychophysical approach to estimate the perceptual detection threshold for spinal curvature using a Bayesian adaptive procedure (Kontsevich and Tyler 1999). In addition, AIS patients completed the Trunk Appearance Perception Scale (TAPS) to correlate objective detection thresholds with subjective self-perception of their own deformity. Furthermore, we compared TAPS scores against patients’ clinically diagnosed Cobb angles to determine whether subjective body schema distortion directly reflects objective structural deformity or operates as a distinct clinical feature. We hypothesized that AIS patients would demonstrate lower perceptual thresholds (higher sensitivity) compared to controls, reflecting an attentional bias and a more sensitive internal representation of the body, resulting in a heightened ability to detect subtle deviations from verticality. In addition, we hypothesized that TAPS scores would correlate strongly with objective visual detection thresholds but would remain independent of the physical severity of the Cobb angle.

In the second task, we assessed the neurophysiological response to these body stimuli by measuring corticospinal excitability. We applied single-pulse TMS over M1 at an early post-stimulus window (100–125 ms) while participants performed a visual detection task featuring spinal curvatures at, above, and below their previously established thresholds. This window allowed us to probe the rapid sensorimotor integration of body-relevant information. We expected participants with AIS to demonstrate higher accuracy in detecting these changes, as their attentional resources are potentially biased towards spinal and postural changes and they are likely to allocate more attention to relevant body parts, notably the spine and trunk.

For individuals with AIS, images depicting spinal curvature are not merely neutral visual stimuli but are highly salient, self-relevant cues that may trigger an automatic motor simulation. Prior evidence suggests that M1 excitability reflects the accumulation of evidence during perceptual decision-making (Hobot et al. 2020). We therefore hypothesize that in AIS, this baseline hyperexcitability acts as a substrate for heightened sensitivity, wherein the ‘tuning’ of the motor system toward spinal morphology will manifest as threshold-specific modulation. Specifically, the motor cortex should reach peak excitability at the exact moment the visual system detects a distortion (the perceptual threshold), whereas healthy controls will not show this specific coupling between perceptual awareness and motor readiness.

## Materials and methods

The experiment consisted of three tasks. Below we describe participant recruitment and selection criteria, visual stimulus creation, experimental procedures, and statistical analyses.

The study was carried out according to the ethical principles of the 1964 Declaration of Helsinki (Declaration of Helsinki, 2013) and was approved by the Ethics committee of the University of Verona (n. 32/2024). Written, informed consent was obtained from all participants.

### Participants

Participants with AIS were recruited from *“Studio Alberti Postura & Movimento”*, located in Verona, Veneto, Italy, and healthy controls were recruited from the University of Verona. All participants provided written informed consent prior to enrollment. To ensure a homogenous clinical sample, inclusion criteria for the patient group required a diagnosis of AIS in early adolescence, confirmed by an orthopaedic specialist. Cobb angle measurements were verified through medical records, which also confirmed the absence of other orthopaedic or neurological conditions involving the spine. Critically, no participants had undergone prior surgical intervention for scoliosis, ensuring that the measured neurophysiological responses reflected the lived experience of the primary deformity rather than post-surgical adaptation.

No participants reported a history of neurological disorders, a documented history of epilepsy, or confirmed seizures. Furthermore, none were taking medications known to interfere with neuronal excitability (Rossi et al. 2021). All participants possessed normal or corrected-to-normal vision, were naïve to the specific purposes of the experiment, and reported or exhibited no discomfort or adverse events associated with the transcranial magnetic stimulation (TMS) protocol.

### The trunk appearance perception Scale (TAPS)

The Trunk Appearance Perception Scale (TAPS) is a validated instrument with high internal consistency and excellent test-retest reliability, developed to evaluate the subjective perception of trunk deformity in individuals with IS (Bago et al. 2010). The scale consists of three sets of figures, each containing five drawings that depict the trunk from three viewpoints: anterior (from the front), posterior (from the back), and during forward bending. Participants are asked to select the drawing in each set that most closely resembles their own trunk appearance. Each drawing is scored on a scale from 1 (greatest perceived deformity) to 5 (least perceived deformity), with higher scores reflecting a more positive self-perception of trunk appearance. The final TAPS score was calculated by averaging the scores from three of the four original drawing sets (Front, Back, and Bending), the male-specific set was excluded as all participants were female (see Supplementary Material 1).

### Visual stimuli

**Fig. 1:**
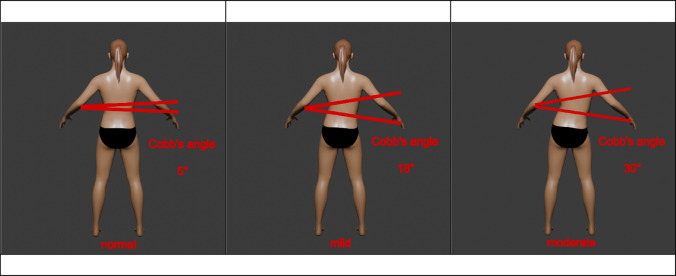
Examples of stimuli for normal, mild, and moderate simulation of thoracolumbar curve. Red markings were added here for illustrative purposes and were not present during stimulus presentation

All stimuli were created using Blender 4.1 (Blender Online Community, 2025) with the MakeHuman plug-in (MakeHuman Community, 2024). The stimuli consisted of images displaying a 3D-rendered female body from a posterior view, presented against a black background. To minimize variability, the same body model was used across images. The images depicted varying degrees of a right thoracolumbar scoliotic curve (see Fig. 1), quantified by the Cobb angle. In this study, stimuli were categorized based on Cobb angle ranges: normal curvature (0° to 11°, *n* = 12), mild scoliosis (12° to 21°, *n* = 10), and moderate scoliosis (22° to 37°, *n* = 16). These cutoffs were selected according to established clinical guidelines commonly used in scoliosis assessment (Horng et al. 2019). Each image was labelled according to its corresponding curvature angle. A total of 38 stimuli were used in the experiment with examples reported in Fig. 1. All images were displayed on a 17-inch monitor positioned approximately at 60 cm from the participants’ eyes.

### Transcranial magnetic stimulation and electromyography recording

Pairs of surface EMG electrodes were placed in a belly-tendon montage over the right first dorsal interosseous (FDI) muscle, with the active electrode over the muscle belly and the ground electrode on the right wrist. Corticospinal excitability was assessed via motor-evoked potentials (MEPs) elicited by biphasic single-pulse TMS (STM 9000, Ates-EBNeuro, Italy) using a 110 mm figure-of-eight coil. The coil was held tangentially to the scalp at a 45° angle to the midline to induce a posterior-anterior current flow in the left primary motor cortex (M1). The optimal stimulation site (the ‘hotspot’) was initially positioned based on the International 10–20 system (approximately 5 cm lateral to Cz) and then functionally identified as the site consistently evoking the maximal MEP amplitude in the relaxed contralateral FDI. This site was marked on the scalp to ensure consistent positioning. Participants’ heads were stabilized using a headrest, and the coil was fixed via an adjustable mechanical arm. The resting motor threshold (rMT) was defined as the lowest intensity required to evoke an MEP of at least 50 µV in the FDI in at least 5 out of 10 consecutive trials (Rossini et al. 2015). During the experimental task, stimulation intensity was set at 120% rMT (Hallett 2000); mean rMT (± SD) was 54.2 ± 4.8% of maximum stimulator output. EMG activity was recorded using a wireless system (Zerowire, Aurion, Italy) and acquired through a Cambridge Electronic Design 1401 (CED, UK) interface. Signals were sampled at 5 kHz and stored for offline analysis. A Scarlett 6i6 audio card (Focusrite, UK) controlled the TMS trigger to ensure synchronicity with stimulus delivery, which was managed via MATLAB 2024b and Psychtoolbox-3 (Brainard and Vision 1997; Kleiner et al. 2007). Ten baseline trials were recorded before the task, with an inter-pulse interval of ~ 10 s to minimize cumulative effects (Hassanzahraee et al. 2019).

### Procedure and experimental design

The experiment took place in a quiet and dimly illuminated room. At the beginning of the experiment, only participants in the scoliosis group were asked to select the drawing in each set of the TAPS questionnaire that most closely resembles their own trunk appearance (Bago et al. 2010). To prevent acute cognitive or attentional priming from influencing subsequent neurophysiological metrics, a gap of approximately 30 min was maintained between TAPS completion and TMS testing. During this interval, participants underwent the physical setup and briefing for EMG electrode placement, which served as a practical time buffer prior to the start of testing. All participants were seated comfortably in front of the computer monitor. The experimental protocol was implemented using a custom MATLAB script which controlled both stimulus presentation and TMS pulse triggering were controlled by a personal computer running MATLAB (MathWorks, MA, USA). The experimental paradigm consisted of two tasks, both of which were completed by all participants. For each participant the overall duration of the experiment took around 90 min.

*Task one: Psychophysical Estimation of Perceptual Thresholds*.

In the first task, participants completed a psychophysical discrimination task to determine their perceptual threshold for detecting spinal curvature abnormality (i.e., the Cobb angle at which the body transitioned from appearing normal to abnormal). Stimulus presentation and response collection were implemented using the Psychtoolbox and the Palamedes MATLAB toolboxes (Prins and Kingdom 2018). An adaptive staircase procedure based on the Psi method (Kontsevich and Tyler 1999) was used to estimate each participant’s perceptual threshold (α) from the psychometric function. A total of 60 images from the designated visual stimuli set were used to establish the individual thresholds. Each trial began with a fixation cross presented for a jittered duration between 0.8 and 1.2 s, followed by the target image. The image remained on screen until the participant responded. Participants judged whether the body appeared “normal” or “not normal” by pressing one of two designated keys (“A” or “L”), with key assignment counterbalanced across participants. They were instructed to respond as quickly and accurately as possible. On each trial, the curvature angle was selected to maximize expected information gain, enabling efficient parameter estimation within a limited number of trials. Invalid responses were flagged. These were defined as key presses occurring within 100 milliseconds of stimulus onset or the use of non-assigned keys. The corresponding trials were administered again at the end of the session. Real-time estimates of threshold and slope, along with their associated standard errors, were recorded after each valid trial.

*Task two: TMS and Motor Evoked Potentials*.

**Fig. 2:**
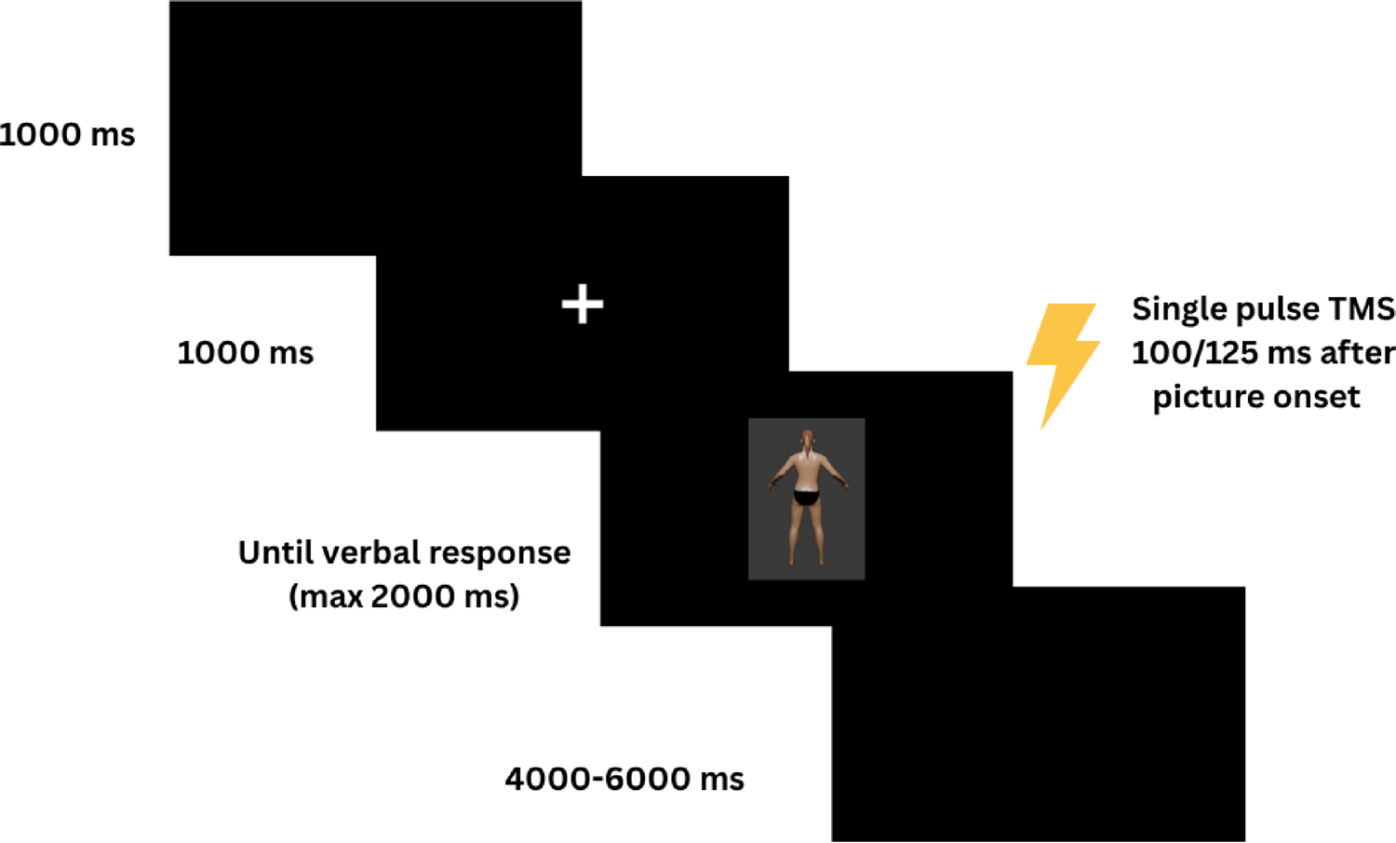
Trial sequence

The second task consisted of 114 trials (38 Cobb angles x 3 repetitions) in which participants again judged whether the female body appeared normal or not. However, in this task, a single pulse of transcranial magnetic stimulation (TMS) was delivered at a fixed latency of 100 ms (or 125 ms) after stimulus onset, with MEPs recorded at these respective time points. Each trial began with a black screen (with a random time between 0.8 and 1.2 s to minimize priming effects), followed by a fixation cross at the center, and then the target image. Participants responded verbally (“yes” or “no”), and the experimenter recorded their responses via keypress on a keyboard. After the response, the image disappeared, and the black screen reappeared for 3–5 s. Additionally, the interval between consecutive trials ranged from 5 to 10 s, reducing the risk of cumulative neural effects from successive TMS pulses (Wassermann et al. 2008). A graphical presentation of the trial structure is presented in Fig. 2.

### Data analysis

#### Behavioural data

For each participant, psychophysical performance was modelled by fitting psychometric functions using maximum-likelihood estimation (Prins and Kingdom 2018). Detection thresholds were extracted from the fitted curves and summarized in Table 1. Threshold data were first assessed for normality using the Shapiro-Wilk test, which indicated that the threshold scores were normally distributed (W = 0.97181, *p* = .867). Based on this, an independent samples t-test was conducted to compare threshold values between groups.

**Table 1:** Mean (± SD) accuracy (%) across conditions for control and scoliosis groups

Condition	Control (*n* = 8)	Patient (*n* = 8)
Normal	96.74 ± 6.90	89.77 ± 11.10
Mild	50.68 ± 18.66	81.67 ± 11.95
Moderate	98.04 ± 2.10	100.00 ± 0.00

Values represent mean percentage accuracy (± standard deviation). Accuracy refers to the percentage of correct responses in the body judgment task

Afterwards, a Pearson correlation analysis was conducted to determine if there is a relationship between self-report measure of body perception (TAPS) and the perceptual threshold within the scoliosis group.

For the second task, accuracy was calculated by comparing participants’ responses against the correct classification for each trial (normal = “yes”, mild/moderate = “no”). Overall accuracy was expressed as the percentage of correct responses across all trials. Condition-specific accuracy was further computed separately for normal, mild, and moderate trials. Accuracy data were assessed for normality within each group and condition using the Shapiro-Wilk test and visualized with Q-Q plots, revealing mixed results with two subsets deviating from normality. Due to repeated measures design and presence of between-subjects (*Group*) and within-subjects (*Condition*) factors, a linear mixed-effects model was employed, incorporating random intercepts for subjects, equivalent to a 2 (Group: *patient*,* control*) × 3 (Condition: *normal*,* mild*,* moderate*) mixed-design ANOVA. Effect sizes for main effects and interactions were computed using partial eta squared (η²_p_ ).

#### Neurophysiological data

Neurophysiological data were recorded and processed offline. EMG signals were band-pass filtered (30–500 Hz) using a second-order Butterworth filter applied in both forward and reverse directions to avoid phase distortion. Trigger channels were filtered (20–2000 Hz) and normalized to identify stimulus onsets by employing ‘findpeaks’ from Signal Processing toolbox in MATLAB. For each trial, EMG epochs were extracted in the 20–50 ms post-stimulus interval. Peak-to-peak MEP amplitudes (µV) were defined as the difference between maximum and minimum EMG values within the identified response window. Trials were reviewed interactively: epochs with clear MEPs were accepted, corrupted or artefactual responses were rejected, and when necessary, the response window was manually adjusted and the peak-to-peak procedure repeated.

Prior to statistical evaluation, outliers were identified per-participant and per-condition using Tukey’s Method (1.5 × interquartile range criterion), resulting in the removal of 3.7% of trials (Tukey 1977). To control for inter-individual anatomical differences, MEP amplitudes were normalized to each participant’s mean baseline MEP (see Sect.  2.4). Normalized values were log-transformed to stabilize variance, then aggregated across repetitions using the mean, yielding one value per participant per condition.

Due to non-normality of residuals (Shapiro-Wilk test, *p* < .001), data were analyzed using aligned rank transform ANOVA (Wobbrock et al. 2011) with Group (2 levels: control, scoliosis) as a between-subjects factor and Condition (3 levels: normal, mild, moderate) as a within-subjects factor. Effect sizes for main effects and interactions were computed using partial eta squared (*ƞ*_*p*_^2^).

To examine group differences in the angle-dependent modulation of corticospinal excitability, we modeled trial-level log-transformed MEP values using locally weighted regression (LOESS; Cleveland et al. 1992) with a second-degree polynomial and smoothing span of 0.75. To reduce the influence of participants with highly variable responses, trials were weighted by the inverse of each participant’s interquartile range of log-transformed MEP values. For this analysis, we applied an alternative normalization by subtracting each participant’s mean log-transformed MEP measured for the stimulus with zero degrees (straight back posture) from all their responses, thereby expressing MEP modulation as relative changes from an upright baseline. Predicted fits and standard errors were computed across non-zero stimulation angles (1–37°) and plotted separately for control and patient groups.

#### Statistical packages

Threshold, accuracy and TAPS statistical analyses were conducted using JASP version 0.19.3 (JASP Team, 2025), whereas for visualization R version 4.5.2. (R Core Team, 2025) with RStudio version 2025.09.02 were used, along with the packages ggplot2 (v4.0.0), dplyr (v1.1.4) and readxl (v1.4.5). MEP analyses were conducted in R version 4.5.2 (R Core Team, 2025) using RStudio version 2025.09.2, with the packages data.table (v1.17.8), ggplot2 (v4.0.0), ARTool (v0.11.2), and stats (v4.5.2) for data processing, visualization, and statistical modelling.

## Results

### Participants

The participants pool consisted of eight female participants diagnosed with adolescent idiopathic scoliosis (age mean (M) = 19.6, standard deviation (SD) = 1.9 years) and eight healthy, age- and sex-matched controls (age M = 22.3, SD = 1.9 years). All participants were right-handed. Within the AIS group, the estimated time since diagnosis was approximately 5 years, and participants had been treated with specialized corrective exercises for an average of 4.6 years (range: 4–6 years). Regarding curve topography, six participants presented with a single primary right thoracolumbar curve, while two participants presented with double curves, characterized by a primary right thoracic curve and a secondary left lumbar compensatory curve. Cobb angle measurements of the main scoliotic curve ranged between 20° and 41°, with a mean Cobb angle of 31.0° (standard error (SE) = 2.5°). No participants had ever undergone conservative treatment with spinal bracing, and none had a history of prior surgical intervention.

### Perceptual threshold

**Fig. 3:**
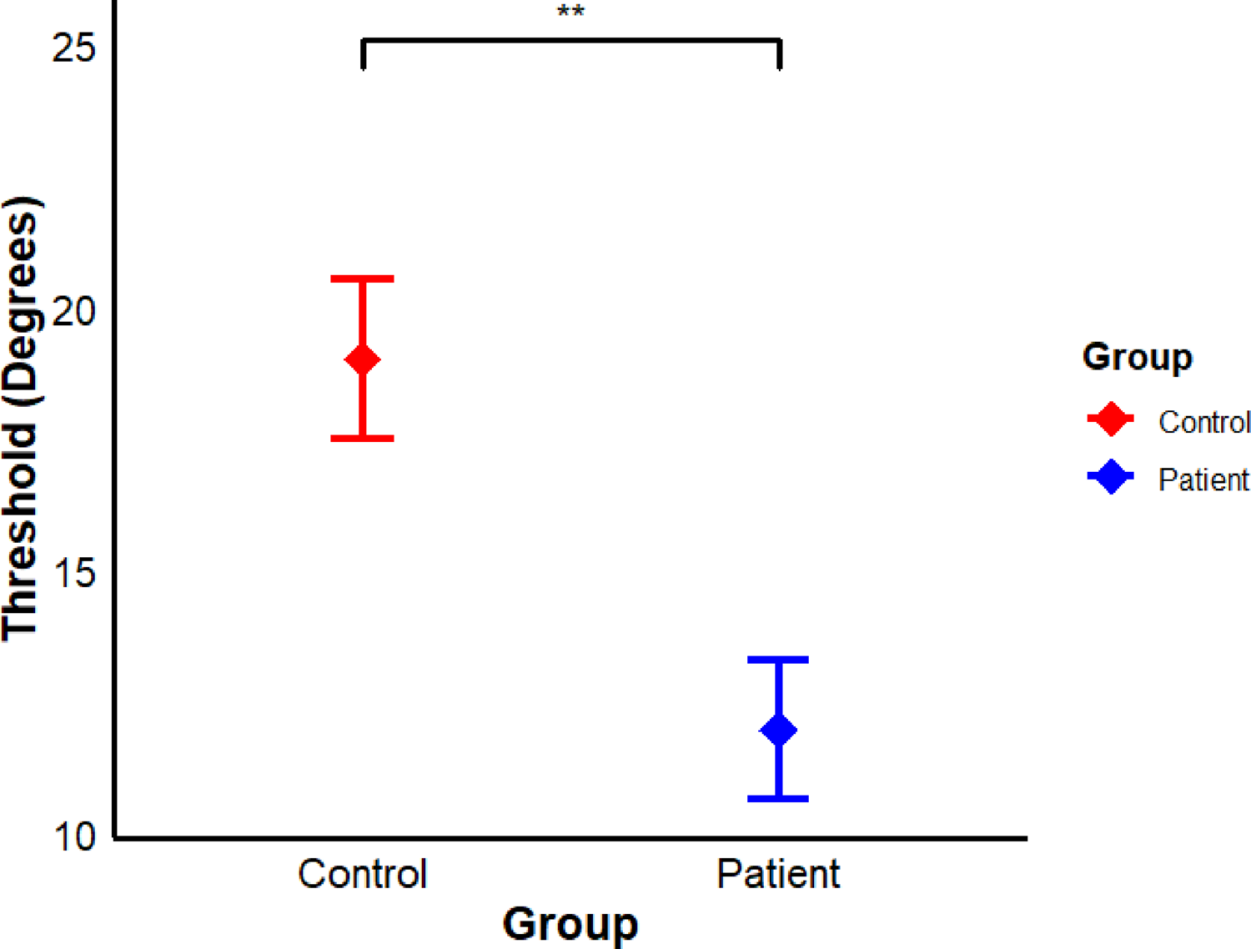
Distribution of threshold degrees across groups. Mean threshold degrees across groups. The patient group demonstrated lower values than the control group, consistent with the significant difference found in the independent samples t-test (t(14) = 3.47, *p* = .004, Cohen’s d = 1.7). Error bars represent ± 1 standard error of the mean (SEM), and ** represents *p* < .01

An independent samples t-test was conducted to compare perceptual thresholds (in degrees) between groups. Threshold scores were significantly lower (*t*(14) = 3.47, *p* = .004, Cohen’s d = 1.74, 95% confidence interval (95%-CI) [0.55, 2.88]) for patients (M = 11.97, SE = 1.34), than for control group (M = 19.02, SE = 1.53). A graphical representation is reported in Fig. 3.

### Correlation analysis of trunk appearance perception (TAPS), perceptual threshold and clinical deformity

Results of the Pearson correlation indicated that there was a strong, significant positive correlation between the TAPS score and perceptual threshold, (*r*(6)= 0.876, *p* = .004, 95% CI [0.446, 0.977]), indicating that a more adequate self-perception of trunk appearance (higher TAPS scores) was associated with a higher perceptual threshold (α).

Instead, Spearman’s rank correlation between participants’ Cobb angles and TAPS scores revealed a non-significant correlation between objective curve severity and subjective body schema distortion (*r*_*s*_ = 0.383, *p* = .349, 95% CI [-0.440, 0.857]).

### Perceptual accuracy

**Fig. 4:**
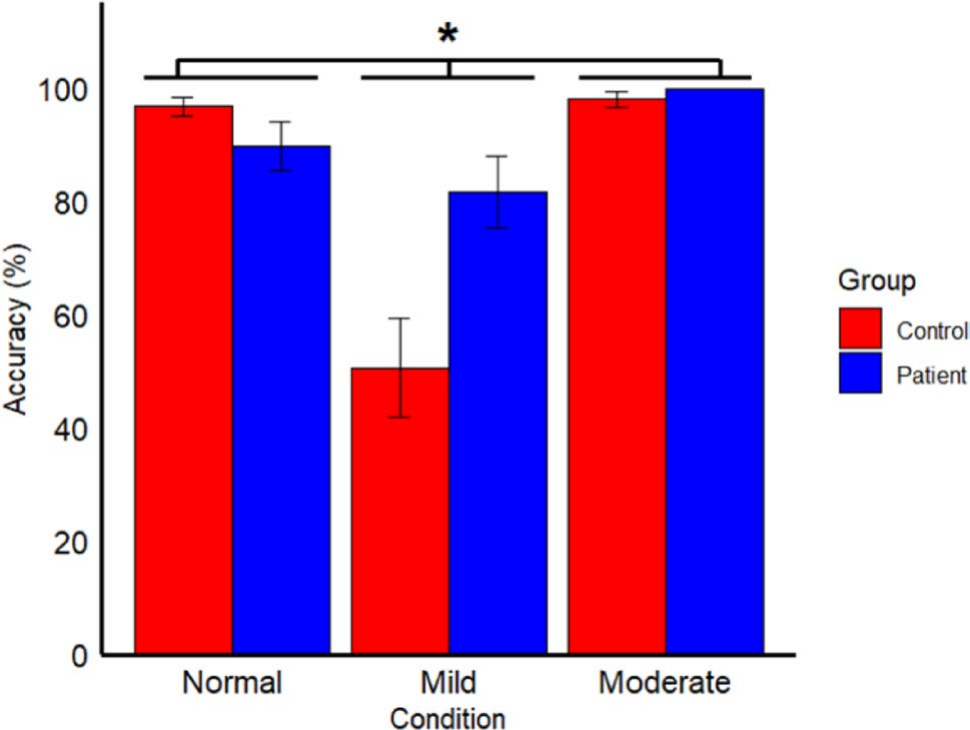
Mean accuracy (%) across conditions. Interaction between Condition (Normal, Mild, Moderate) and Group (Patient vs. Control). Error bars represent ± 1 standard error of the mean (SEM), and * represents *p* < .05

A mixed-design ANOVA was conducted to compare accuracy in judging body distortions (*Group* as a between-participant factor with levels *patient* and *control*, and *Condition* as a within-participant factor with levels *normal*, *mild* and *moderate*). Mauchly’s test indicated that the assumption of sphericity was violated for the within-subjects factor, *Condition* (χ^2^(2) = 21.08, *p* < .001). Therefore, Greenhouse–Geisser corrections (ε = 0.56) were applied. The analysis revealed a significant main effect of *Condition* (F(1.11, 15.53) = 34.54, *p* < .001, eta η²_p_ = .71) and a significant main effect of *Group* (F(1, 14) = 18.71, *p* < .001, η^2^_p_ = .44). These effects were qualified by a significant *Condition × Group* interaction (F(1.11, 15.53) = 11.06, *p* = .004, η^2^_*p*_ = .44). Follow-up analyses showed no group difference in the *normal* condition (F(1, 14) = 2.28, *p* = .154), but significant group differences in both the *mild* condition (F(1, 14) = 15.65, *p* = .001), and the *moderate* condition (F(1, 14) = 7.00, *p* = .019), with the *patient* group showing higher accuracy. Descriptive statistics are presented in Table 1.(Figure 4)

### Neurophysiological data

**Fig. 5:**
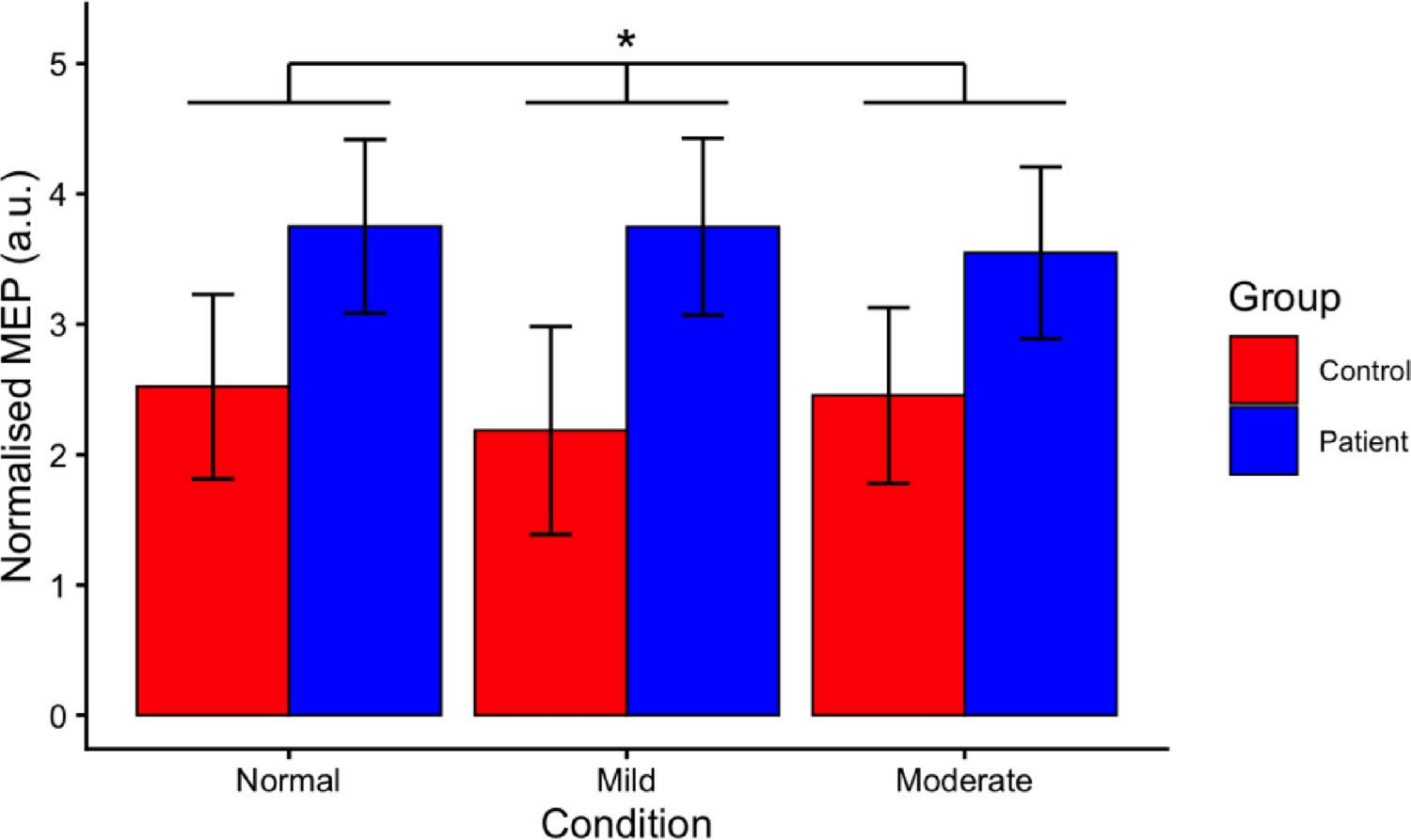
Group differences in normalized and transformed motor evoked potentials (MEP) across condition levels. Normalization was implemented by dividing each MEP amplitude by the participant’s resting motor threshold baseline, followed by log-transformation. Bars display mean log-transformed MEP values for Control and Patient groups across Normal, Mild, and Moderate conditions. Ranges represent standard errors, and * represents *p* < .05

Data were analysed using aligned rank transform ANOVA with *Group* (levels: *control*,* patient*) as a between-subjects factor and *Condition* (levels: *normal*,* mild*,* moderate*) as a within-subjects factor. The analysis revealed a significant main effect of *Group* (F(1, 42) = 8.29, *p* = .006, η²_p_ = .16), indicating overall larger MEP responses in the *patient* group compared to controls. The main effect of Severity was not significant (F(2, 42) = 0.11, *p* = .900, η^2^_p_ = .005), nor was the *Group* × *Condition* interaction (F(2, 42) = 0.17, *p* = .844, η^2^_p_ = .008). Post-hoc pairwise comparisons on the aligned rank-transformed data, with patients showing significantly higher log-transformed MEP values (M = 3.68, SE = 0.40) than controls (M = 2.39, SE = 0.37; contrast estimate = − 11.2, SE = 3.91, t(42) = − 2.88, *p* = .006). This result, visualized in Fig. 5, demonstrates that patients exhibited consistently greater corticospinal excitability than controls, independent of severity level as observed in previous literature (Boček et al. 2022; Doménech et al. 2010).

**Fig. 6:**
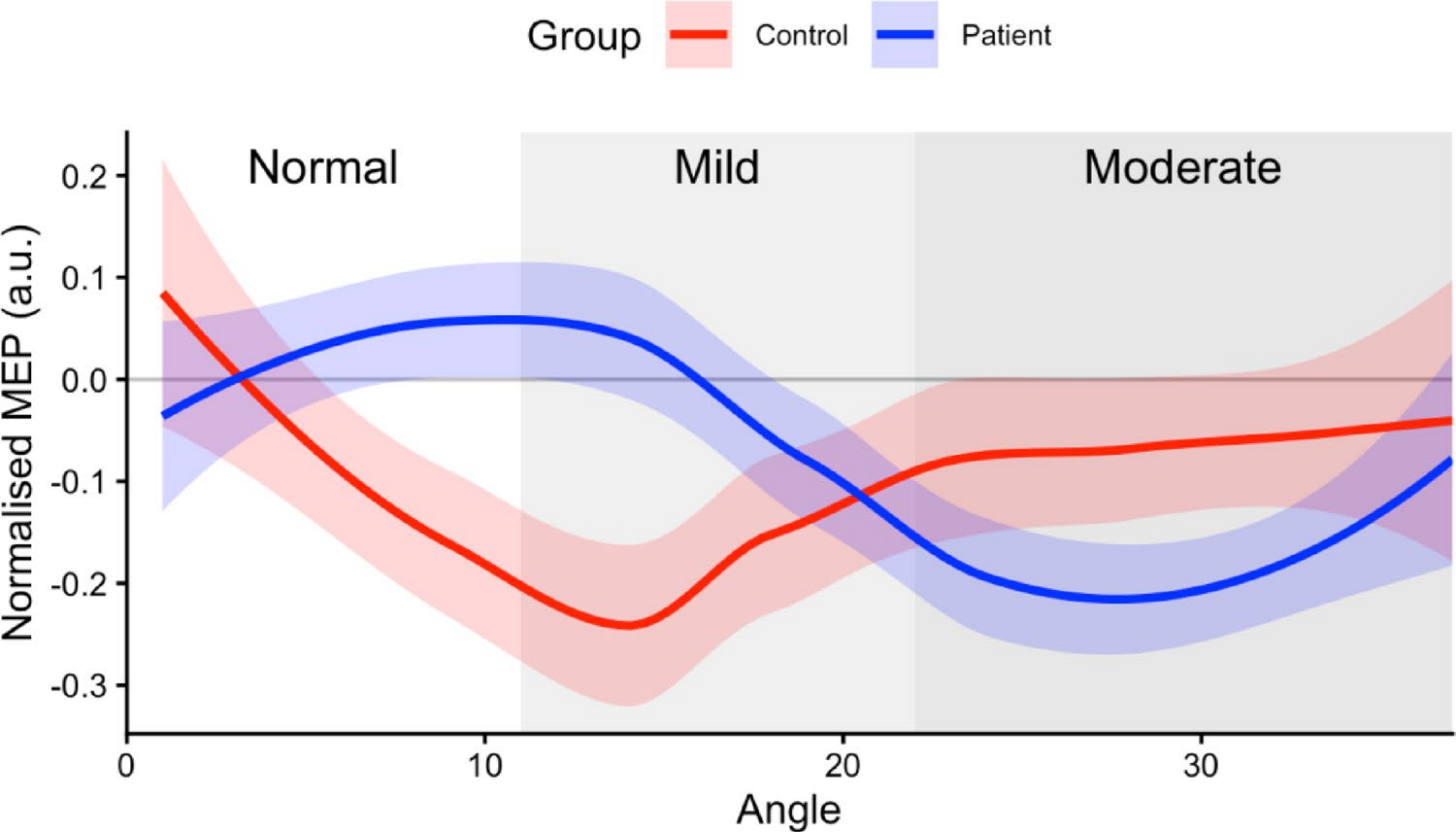
Angle-dependent modulation of corticospinal excitability relative to upright baseline. Locally weighted regression curves show log-transformed MEP amplitudes normalized to each participant’s response at 0° (straight back posture, excluded from the figure) as a function of stimulation angle. Individual trials were weighted by the inverse of participant-level interquartile range to reduce the influence of highly variable responses. Solid lines represent fitted values for Control (red) and Patient (blue) groups; shaded ribbons indicate ± 1 standard error. Background shading denotes condition regions: Normal (0–11°), Mild (11–22°), and Moderate (22–37°)

Figure 6 illustrates distinct group-specific angle-dependent non-linear patterns that align with perceptual thresholds. MEP values are normalized to each participant’s response at 0° (straight back). Patients showed peak excitability around ~ 10.5 degrees, matching their lower perceptual threshold (M = 11.97°), indicating maximal motor engagement at their perceptual decision boundary. Excitability then progressively declined toward moderate angles, where severely curved spines visually resemble their own body configuration (participants Cobb angle M = 31.0, SE = 2.39) and demonstrated to be less salient. Controls showed minimal excitability near ~ 14 degrees, reflecting that these angles remain below their higher perceptual threshold (M = 19.02°) and are processed as normal, requiring little motor system engagement. Their excitability gradually increased toward moderate angles as curvature exceeded their detection threshold and became clearly abnormal. The smaller difference in the moderate range reflects that for patients, severe curvatures are familiar and less engaging, whereas for controls, these angles represent clear abnormalities. These opposing patterns provide neurophysiological evidence that motor cortex excitability connects to perceptual salience: patients engage maximally at their expertise boundary, while controls engage minimally until curvature becomes obviously abnormal.

## Discussion

An accurate perception of one’s own body is fundamental to sensorimotor control, a stable self-image, and body awareness. Previous research has demonstrated that this perceptual model depends on the integration of visual, proprioceptive, tactile, vestibular, and interoceptive inputs from distributed cortical networks (Palermo et al. 2014; Tsakiris 2017). Consequently, modifications to any sensory pathway can alter body perception. This phenomenon has been reported in numerous conditions, including adolescent idiopathic scoliosis (AIS). While previous research has predominantly emphasized proprioceptive deficits, our results suggest that AIS patients display increased visual-perceptual sensitivity to spinal deformity, potentially reflecting a functional sensory compensation for altered underlying proprioceptive inputs.

The results of the first task showed that AIS patients exhibited significantly lower perceptual thresholds than controls, indicating hypersensitivity to subtle spinal distortions. This aligns with reported alterations in postural perception and sensory integration in AIS (Assaiante et al. 2012; Paramento et al. 2024). Paralleling the “expert” motor systems of athletes (Aglioti et al. 2008), the continuous sensorimotor experience of spinal asymmetry in AIS likely induces long-term potentiation (LTP)-like plasticity. This plastic adaptation may heighten corticospinal gain, effectively priming the primary motor cortex to react to subtle morphological deviations that fall below the perceptual threshold of healthy observers. Such heightened neural gain suggests that AIS involves a specialized, rather than deficient, mode of perceptual-motor integration.

Additionally, we found a strong positive correlation between patients’ self-perception of their trunk appearance (as measured by TAPS; Bago et al. 2010) and their perceptual threshold. This suggests that patients who perceive their bodies more positively (higher TAPS scores) require larger distortions to detect abnormalities. Conversely, patients with a negative self-perception of their own body (lower TAPS scores) have an extremely low threshold and can detect subtle body distortions. This links subjective body perception directly to objective perceptual sensitivity. Specifically, it can be suggested that the subjective experience of deformity is a robust top-down modulator that lowers the visual system’s threshold for detecting deformation.

Crucially, our secondary analysis revealed that TAPS scores were not significantly correlated with participants’ Cobb angles (see Sect.  3.3). Such lack of connection between objective curve severity and subjective perception might suggest that body image disturbance is a prominent, independent neurophysiological feature that can manifest profoundly even in those with mild scoliosis (Belli et al. 2022). However, given our limited sample size (*N* = 8), this lack of statistical significance must be interpreted with caution. These findings tentatively suggest that rather than being a passive reflection of scoliotic curve, the internal body schema appears to operate as a centralized sensorimotor phenomenon. This potential independence suggests that chronic, asymmetrical proprioceptive inputs from the scoliotic spine drive a top-down reorganization of the body schema over time. Consequently, patients’ sensory vigilance may operate independently from the actual physical severity of their scoliosis.

In the second task, patients demonstrated greater accuracy in identifying spinal curvature distortions, compared to healthy controls, consistent with our expectations. This increased accuracy likely reflects the heightened attention and sensitivity to the body posture, already established in the first task. Because patients with AIS frequently monitor and compare their own posture to that of others, they may develop a form of hypervigilance towards spinal alignment. Prior research supports this interpretation, showing that AIS patients exhibit a specific attentional bias toward body parts affected by this pathology, resulting in higher task-specific accuracy (Bertuccelli et al. 2023). This selective bias can account for their superior performance relative to healthy controls, who tend to distribute attention more evenly across stimuli. On the contrary, controls may perceive subtle spinal distortions as low-priority visual cues and thus allocate less attention which in turn results in lower accuracy.

Interestingly, the greatest performance difference between the patients and controls emerged in the mild curvature condition (Cobb’s angle 12°-21°). Specifically, in this range, the control group demonstrated markedly reduced accuracy (51%), suggesting that such subtle changes fall below the detection threshold for the average observer, whereas patients maintained high accuracy (82%) in detecting these distortions. This pattern supports the idea that patients with AIS develop a specialized sensitivity to spinal asymmetry within the range that defines their pathology (> 11° Cobb’s angle). In line with the attentional priority framework (Fecteau and Munoz 2006), subtle spinal curvature cues may be assigned low priority by healthy controls in the absence of personal or disease-specific relevance, resulting in reduced allocation of attention and lower accuracy.

Confirming our primary hypothesis, AIS patients exhibited significantly greater overall corticospinal excitability than controls, regardless of curvature severity. This suggests heightened sensorimotor engagement and increased corticospinal gain during body-related visual processing (Borgomaneri et al. 2014). Since M1 is functionally integrated with parietal networks governing body schema, MEPs recorded from the FDI serve as a sensitive readout of this systemic activation. Although recorded distally, resting-muscle M1 excitability during visual tasks reflects the global state of motor readiness. Given the functional coupling between axial and distal motor representations in postural maintenance (Chiou et al. 2016), these MEPs provide a reliable window into the systemic sensorimotor recalibration occurring in AIS, where the motor system is “primed” by body-relevant stimuli.

While MEPs measure corticospinal excitability, body representation is a multimodal construct shaped by visual-to-motor mapping (Cesari and Urgesi 2018). In AIS patients, the visual input of a scoliotic body likely engages these integrated pathways, triggering a heightened embodied simulation. Given the specialized cortical reorganization in this population, viewing spinal distortions appears to elicit a primed motor response, reflected by significantly higher MEP amplitudes. Rather than viewing this motor cortical hyperexcitability as an exclusive consequence of structural pathology or a purely functional perceptual mechanism, our findings point toward a complex interplay between the two. The structural pathology of AIS (associated with altered white-matter pathways and spinal deformity) likely establishes a chronically elevated baseline of corticospinal excitability, essentially a state of neural ‘readiness.’ However, this baseline is dynamically modulated by top-down visual processing related to body image and self-relevance. When patients view body-related visual distortions, the underlying hyperexcitability acts as a sensitive sensorimotor amplifier, resulting in the threshold-specific coupling observed during perceptual awareness. Thus, the heightened excitability reflects both the persistent structural pathology and the functional demands of processing salient, body-specific visual inputs.

However, differences between patients and controls may also reflect a specific reorganization of the sensorimotor system in AIS. From this perspective, the findings can be interpreted as evidence of adaptive sensitivity within the motor system, suggesting that individuals with AIS possess a distinct, rather than deficient, mode of perceptual-motor integration. Our analysis of corticospinal excitability across different Cobb angles revealed non-linear modulation patterns that point to a functional ‘tuning’ of the motor system. When MEP values were normalized to each participant’s response at 0° (straight back), the resulting pattern was directly coupled to individual perceptual boundaries, suggesting that the motor system is specifically calibrated to detect the onset of spinal deviation.

Specifically, patients showed peak corticospinal excitability at approximately 10.5° of spinal curvature, closely matching their fine-tuned perceptual detection boundary (M = 11.97°). This peak represents maximal motor system engagement at the perceptual decision point, with motor engagement progressively declining as curves became more visually pronounced and entered the moderate range. This finding is particularly noteworthy given that the average Cobb angle of the patients in our study was 31° (range 20° − 41°), meaning their actual scoliotic curves predominantly fall within moderate scoliosis range. Thus, maximal compensatory engagement appears to occur when spinal deformation is subtle or ambiguous, rather than when it is overt.

In contrast, healthy controls showed minimal excitability for curves around 14°, suggesting that deviations below their higher perceptual threshold (M = 19.02°) are categorized as non-salient. Motor cortex engagement increased only once the visual stimuli exceeded the group’s detection boundary, indicating that the motor system is primarily recruited when curvature is perceived as clearly pathological. These findings align with prior research showing that neural activation during the observation of disordered movement is shaped by an observer’s prior expertise and visual experience with specific motor patterns (Fiorio et al. 2010). Accordingly, controls, who lack familiarity with spinal distortions, show maximal corticospinal activation when viewing clearly pathological curvature, whereas AIS patients show maximal excitability as soon as curvature begins to deviate from normal spine alignment, reflecting a motor system finely tuned by internal body-schema representation.

These findings suggest significant therapeutic potential for interventions targeting perceptual-motor adaptation. By leveraging the nervous system’s capacity for reorganization, such training could improve body representation and functional outcomes in AIS. While the current study provides valuable insights into the neurophysiological underpinnings of body schema in AIS, several limitations must be acknowledged.

Firstly, the present study is limited by its small sample size (*n* = 8 per group), therefore caution is needed when generalizing these non-linear excitability trends. Within our AIS sample, the mean time since diagnosis was 5 years. Over extended periods, chronic asymmetrical proprioceptive and inputs from a deformed spine can drive progressive cortical reorganization and central sensitization. As previously noted, neuroimaging evidence confirms that these prolonged asymmetries correspond with structural alterations in central white-matter pathways (Paramento et al. 2024). This time-dependent sensitization may explain individual variances in TAPS scores and neurophysiological responses, as the internal body schema becomes increasingly fixed or altered over years of lived experience with the primary deformity. Future longitudinal research featuring larger, heterogeneous cohorts should explicitly control for the time elapsed since diagnosis to isolate how the chronicity of scoliosis shapes progressive cortical adaptation.

Another limitation is the heterogeneity of the scoliotic curves within our sample. Although all patients shared a consistent right-sided primary curve, six presented with single thoracolumbar curves, whereas two presented with double structural curves (primary right thoracic with secondary left lumbar compensatory curves). Clinical and biomechanical evidence demonstrates that body image perception instruments, such as the TAPS, fluctuate significantly depending on the precise anatomical location and complexity of the spinal deformity. Thoracic, thoracolumbar, and double curves distort the body silhouette in highly distinct patterns, which likely project unique, altered proprioceptive and somatosensory inputs to the cortex. Because our limited sample size prevented a stratified sub-analysis, future research utilizing larger cohorts is necessary to systematically isolate how specific scoliosis curve types and structural configurations independently drive cortical reorganization and body schema distortion.

Furthermore, the nature of conservative treatment within our sample must be considered. None of the AIS participants in our sample had undergone spinal bracing, a treatment modality known to drastically alter tactile and exteroceptive inputs to the cortex over extended periods. Instead, our participants were treated exclusively with specialized corrective exercises. While bracing creates a passive, external sensory, active exercise treatment continuously recruits and modifies proprioceptive pathways and frequently incorporates visual feedback systems (e.g., mirror training) to recalibrate body perception (Kuru et al. 2016). Because our sample was homogeneous in its exclusive exposure to exercise, our neurophysiological findings reflect a system shaped by active sensorimotor rehabilitation rather than passive mechanical restriction. Nonetheless, future studies should directly compare braced versus exercise-only samples to systematically isolate how distinct conservative treatments differentially impact cortical reorganization and body schema.

Beyond individual physical metrics, the broader psychosocial ecosystem of the patient should also be considered. Clinical everyday practice shows that sensitization to spinal deformities is not exclusive to the patient but frequently extends to the family unit, particularly parents. Specifically, parents often foster significantly higher levels of concern and heightened anxiety regarding the spinal deformity compared to the adolescent patients themselves (Bridwell et al. 2000). Persistent parental hyper-vigilance or anxiety regarding the scoliotic curve can act as a chronic external stressor that may maladaptively reinforce the patient’s own central sensitization pathways. While the current study did not quantify parental dynamics, future investigations should explore the relationship between parental perception of deformity and the patient’s own cortical sensitization profiles.

Finally, while static stimuli effectively probed the internal body schema, future studies utilizing dynamic stimuli or virtual reality would better capture the ecological postural challenges of AIS. Finally, longitudinal research is required to determine the causality between subjective body image (TAPS) and objective perceptual sensitivity.

## Conclusion

In summary, our study reveals a profound neurophysiological link between body perception and motor activation in AIS. Patients exhibit heightened visual sensitivity to spinal deviations, which is directly modulated by their subjective body schema. Crucially, corticospinal excitability in AIS is not linearly related to deformity severity but instead peaks at the individual’s perceptual detection threshold. This suggests that the motor system in AIS undergoes experience-dependent reorganization, characterized by an increased corticospinal gain specifically coupled with the perception of spinal asymmetry. These findings provide a neurobiological basis for incorporating perceptual-motor training into the clinical management of adolescent scoliosis.

## Supplementary Information

Below is the link to the electronic supplementary material.

Supplementary Material 1

## Data Availability

Raw data are publicly available: https://osf.io/y5shp/

## Materials availability

All study materials supporting this research are publicly available: https://osf.io/y5shp/

## Code availability

All analysis code supporting this research is publicly available: https://osf.io/y5shp/

## Declarations

### Design

This article reports, for all studies, how the author(s) determined all sample sizes, all data exclusions, all data inclusion and exclusion criteria, and whether inclusion and exclusion criteria were established prior to data analysis.

### Pre- registration

No part of the study procedures was preregistered in a time-stamped, institutional registry prior to the research being conducted. No part of the analysis plans was pre-registered in a time-stamped, institutional registry prior to the research being conducted.

### Open practices section

Materials, data and code scripts can be accessed via this link to an OSF repository: https://osf.io/y5shp/.
