# Supplementary material for: Motor cortex excitability during spine shape-judgment in adolescent idiopathic scoliosis: a TMS motor evoked potential study
Source: Exp Brain Res. 2026 Jun 30;244(8):147. doi: 10.1007/s00221-026-07343-5 (PMC13319178; doi:10.1007/s00221-026-07343-5)
Supplement: Supplementary file 1 — Supplementary Material 1 [file 221_2026_7343_MOESM1_ESM.docx]

**Supplementary Material**

**Article Title:** *Motor Cortex Excitability in Early Adult Females with Adolescent Idiopathic Scoliosis during Spine Shape-Judgment: A TMS Motor Evoked Potential Study*

**Supplementary Material 1**: Trunk Appearance Perception Scale (TAPS) (Bago et al., 2010).

Scoring Instructions: Each drawing is scored on a 5-point scale, ranging from 1 (greatest perceived deformity) to 5 (least perceived deformity). In the provided sets, the far-right image represents a score of 1, while the far-left image represents a score of 5. The final TAPS score represents the mean of the three included items.


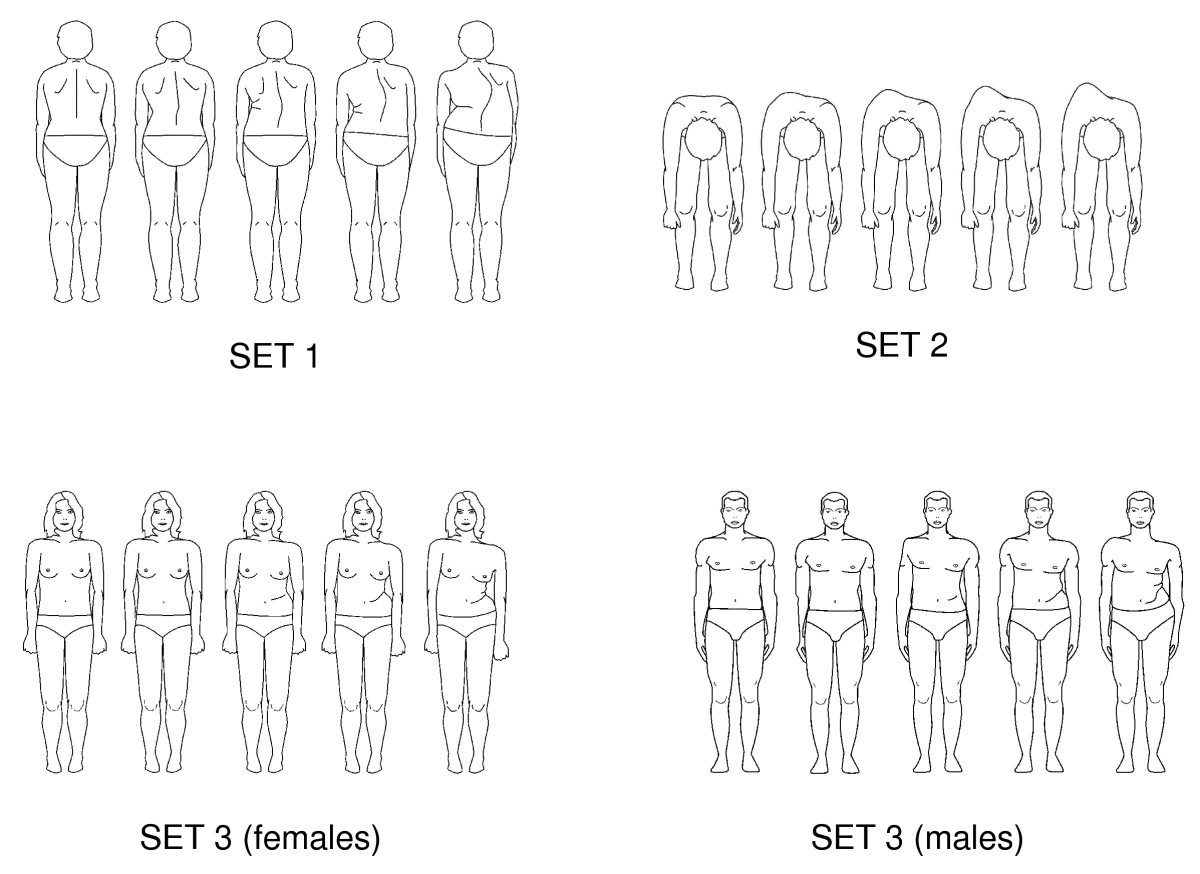
Methodological Note: To maintain the demographic relevance of the stimuli, the male-specific drawing sets were omitted from the presentation as all participants in the current study were female.
